# Komplikationen der 180-W-XPS™-GreenLight-Laserung – Ergebnisse bei 1283 Prozeduren

**DOI:** 10.1007/s00120-022-01988-0

**Published:** 2022-12-13

**Authors:** F. Fallahi, M. Fallahi, R. Brauckmann, S. Brandt, J. Horstmann, A. Wiedemann

**Affiliations:** 1 Urologische Klinik, Ev. Krankenhaus Witten gGmbH, Pferdebachstr. 27, 58455 Witten, Deutschland; 2 Lehrstuhl für Geriatrie, Lehrstuhl für Urologie, Universität Witten/Herdecke, Witten, Deutschland; 3 Urologische Praxisklinik/Zentrum Euregio Franziskushospital Aachen, Aachen, Deutschland; 4 Chrestos Institut, Concept GmbH & Co. KG, Essen, Deutschland

**Keywords:** Photovaporisation der Prostata (PVP), Benignes Prostatasyndrom, Harnverhalt, Infekte, Geriatrischer Patient, Antikoagulation, Photovaporisation of the prostate (PVP), Benign prostate syndrome, Urinary retention, Infects, Geriatric patients, Anticoagulation agents

## Abstract

**Hintergrund:**

Die vorliegende Untersuchung beschäftigt sich mit der Komplikationsrate des Verfahrens in einer neuen Methodik. Es wurden alle Patienten, die innerhalb von 3 Monaten nach ihrer 180-W-XPS™-GreenLight-Laserung (American Medical Systems, Minnetonka, MN, USA) (GLL) erneut stationär aufgenommen wurden, erfasst.

**Methodik:**

Es konnten 170 Patienten ermittelt werden, welche nach ihrer 180-W-XPS™-GLL der Prostata innerhalb von 3 Monaten erneut stationär aufgenommen wurden. Alter, ASA-Score, Antikoagulation, der Umfang der 180-W-XPS™-GLL der Prostata (in Joule) und die Wiederaufnahmegründe in den Kategorien Hämaturie, Harnverhalt, Inkontinenz und Infektgeschehen wurden bei diesen Patienten analysiert und den Patienten ohne Wiederaufnahme gegenübergestellt.

**Ergebnisse:**

Es ergab sich eine Wiederaufnahmequote von 13,25 %. Der Aufnahmegrund war am häufigsten ein Harnverhalt mit 50,6 % und eine Hämaturie mit 49,4 % aller Patienten. Von den Patienten mit einer auftretenden Hämaturie standen 86,75 % unter blutverdünnender Medikation.

**Schlussfolgerung:**

Im Vergleich mit der Referenzzulassungsstudie („Goliath-Trial“), die 135 multizentrische Patienten umfasste, von welchen 14,07 % mindestens eine Grad-II-Komplikation nach Clavien-Dindo erlitten, zeigte sich eine vergleichbare Komplikationsrate. Dies ist umso erstaunlicher, da die mit der GLL in der vorliegenden Untersuchung behandelten Patienten sowohl im Alter als auch mit ihrer in der ASA-Klassifizierung (American Society of Anesthesiologists) ablesbaren Multimorbidität und damit nachfolgenden Polypharmazie Charakteristika geriatrischer Patienten aufweisen. Um Erfolge langfristig postoperativ für diese Patienten sicher stellen zu können, sollte die Verzahnung des ambulanten und stationären Sektors noch optimiert werden.

## Abstract (en)

**Background:**

The aim of this study was to classify and analyze postoperative complications under a new inspection method. This study assessed all patients who presented to the urologic ward within the first 3 months after their surgery.

**Methods:**

A total of 170 patients suffered from complications due to the surgery; 29 patients experienced complications that were not directly correlated with the surgery. Age, American Society of Anesthesiologists (ASA) score, the emitted laser energy of the 180 Watt XPS™ GreenLight laser measured in joules, and the complications which led to the renewed hospitalization such as hematuria, urinary retention, and infection were analyzed and compared in the two groups of patients.

**Results:**

Most complications that occurred were urinary retention and hematuria (50.6% and 49.4% of all patients, respectively). Of the patients with hematuria, 86.75% were under anticoagulant therapy medication.

**Conclusion:**

Compared with the reference approval study (Goliath trial) that included 135 multicentric patients (14.07% of patients presented with a complication classified as Clavien-Dindo II), the rate of complications was similar. This outcome is surprising because the patients treated with the GreenLight laser in the present study were classified as geriatric patients not only according to their age but also in their ASA score which is an indication of multiple other pathologies and administered medications. To achieve long-term postoperative success for these patients, the interface between the ambulatory and the hospital care should be optimized.

**Graphic abstract:**

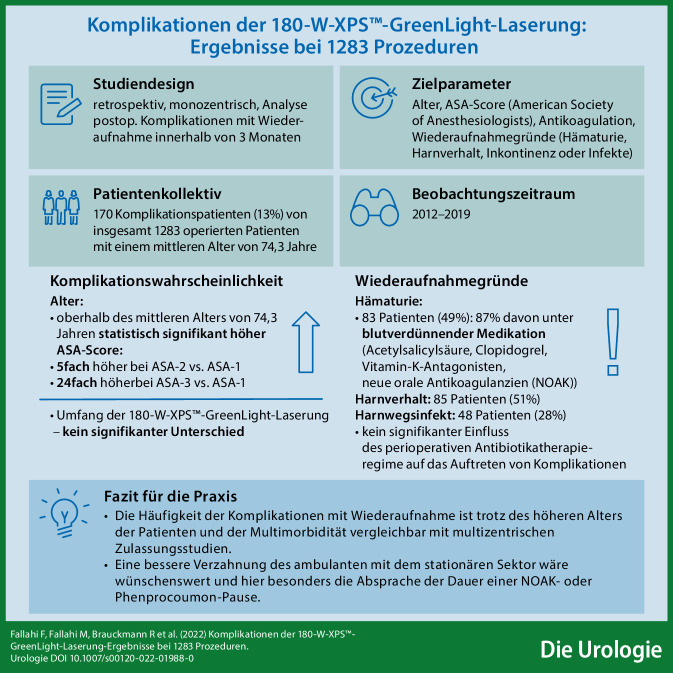

Die 180-W-XPS™-GreenLight-Laserung (American Medical Systems, Minnetonka, MN, USA) (GLL) der Prostata hat ihren festen Stellenwert in der modernen Urologie weltweit. Im Zeitraum 2012–2019 wurden in der Urologie des Evangelischen Krankenhauses in Witten 1283 Patienten mit der 180-W-XPS™-GLL der Prostata behandelt. Die vorliegende Untersuchung beschäftigt sich mit der Komplikationsrate des Verfahrens in einer neuen Methodik unter Real-life-Bedingungen. Es wurden in dieser Untersuchung alle Patienten, die innerhalb von 3 Monaten nach ihrer 180-W-XPS™-GLL erneut stationär aufgenommen wurden, erfasst.

## Einleitung

In der S2e-Leitlinie zur Therapie des benignen Prostatasyndroms (BPS) sind stadienangepasste Behandlungsmöglichkeiten von medikamentösen Konzepten, dem kontrollierten Zuwarten, bis hin zu operativen Eingriffen genannt. Hierzu gehören die als Goldstandard bekannte transurethrale Resektion der Prostata (TURP) und die offene operative Adenomektomie [6, 7]. Daneben kommen verschiedene Lasersysteme, darunter die 180-W-XPS™-GLL zum Einsatz.

**Figure 1:**
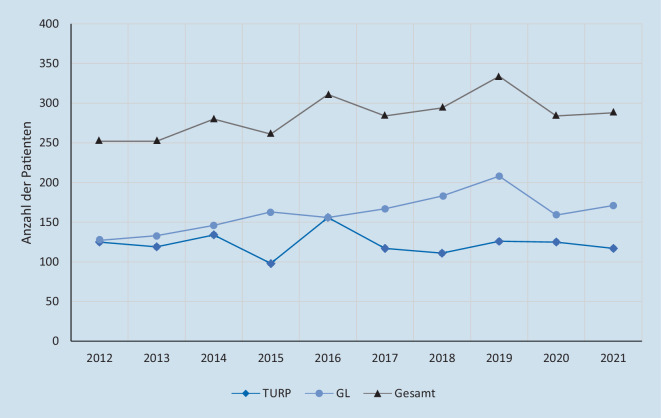

Internationale, multizentrische Studien bestätigten die Gleichwertigkeit hinsichtlich der Sicherheit und Effizienz des 180-W-XPS™-GLL gegenüber der TURP. Ebenfalls bezüglich operativer Einflussfaktoren wie Komplikationen, Nebenwirkungen und Rezidiven sowie patientenbezogener Parameter wie Einfluss auf die Symptomatik, Veränderungen von Prostatavolumen, maximalem Harnfluss und Restharnvolumen war die GLL gegenüber der TURP nicht unterlegen [2]. Patienten, die mit der XPS™-GLL behandelt wurden, erlebten eine deutlich verkürzte Verweildauer, kürzere Katheterliegezeiten und eine schnellere Genesung. Außerdem ist das Verfahren unter Berücksichtigung dieser Faktoren die kostengünstigere Behandlungsoption gegenüber einer TURP [1–3]. Die Komplikations- bzw. Reinterventionsrate des Verfahrens bedarf einer besonderen Einordnung, weil mit der 180-W-XPS™-GLL der Prostata häufig multimorbide, „geriatrische“ Patienten behandelt werden. Aufgrund des hohen Anteils dieser gebrechlichen Patienten, welche unter Blutverdünnung wie z. B. mit Cumarinen, mit neuen oralen Antikoagulanzien oder Thrombozytenaggregationshemmern stehen [4], aber auch wegen des höheren Patientenkomforts hinsichtlich Faktoren wie Schmerz und Spülintensität und des geringeren Personaleinsatzes [5], hat sich das Verfahren in der Abteilung des Ev. Krankenhauses Witten zu dem hausinternen Standard als primäres Verfahren bei der endourologischen operativen Behandlung des BPS entwickelt. Seit 2012 wurden insgesamt 1671 Patienten mit der 180-W-XPS™-GLL der Prostata behandelt (s. Abb. 1).

## Material und Methoden

In dieser Studie wurden retrospektiv 1283 Patienten untersucht, die sich im Zeitraum von 2012 bis 2019 im Ev. Krankenhaus Witten einer operativen Behandlung aufgrund ihrer symptomatischen Prostatavergrößerung mittels einer 180-W-XPS™-GLL unterzogen. Alle Patienten, die innerhalb von 3 Monaten erneut in die Urologie des Ev. Krankenhauses Witten aufgenommen wurden, wurden als sog. „Komplikationspatienten“ erfasst. Dabei erfolgte ein Ausschluss der Patienten, die wegen eingriffsunabhängiger Erkrankungen in diesem Zeitraum hospitalisiert wurden. Die so ermittelten Patienten mit Wiederaufnahme wegen einer Komplikation wurden in ihren demographischen und klinischen Daten denen ohne Wiederaufnahme gegenübergestellt. Erfasste Parameter bei allen Patienten waren das Alter, der ASA-Score und der Umfang der 180-W-XPS™-GLL der Prostata (gemessen mit der Gesamtenergieabgabe in Joule). Nur bei den Komplikationspatienten wurden die Wiederaufnahmegründe in die Kategorien Hämaturie (Patienten mit/ohne Blutverdünner in der Einordnung Marcumar, Heparin, NOAK, TAH), Harnverhalt, Inkontinenz (Dranginkontinenz, Belastungsinkontinenz, Mischinkontinenz), Infektgeschehen und andere Aufnahmegründe erfasst. Patienten mit einem Infektgeschehen wurden separat in den Zeiträumen von 2012 bis 2018 und denen aus 2019 gegenübergestellt. Dies hatte seinen Grund in einer geänderten Antibiotikaprophylaxestrategie: Anfang 2019 war die perioperative Antibiotikatherapie von Ciprofloxacin 2 × 250 mg beginnend ab dem Operationszeitpunkt bis zum 5. postoperativen Tag auf eine reine Single-shot-Prophylaxe mit Cefuroxim 1,5 g i.v. verändert worden. Es sollte überprüft werden, ob sich eine Tendenz bei der Inzidenz entzündlicher Komplikationen nach dem Eingriff erkennen lässt.

Für die stetigen Merkmale wurde der Mittelwert und Median berechnet und zur Beurteilung der Streuungswerte wurde die Interquartilspanne bzw. die Standardabweichung angegeben. Um die Unterschiede der Patientengruppen zu beurteilen, wurden der Wilcoxon-Mann-Whitney-Test und der exakte Fisher-Test sowie logistische Regressionsanalysen durchgeführt. Der *p*-Wert der Teste wurde jeweils deskriptiv zum lokalen 5 %-Signifikanzniveau beurteilt.

## Ergebnisse

### Demographische Daten

Von insgesamt 1283 behandelten Patienten wurden 170 Patienten oder 13,25 % mit einer Komplikation ihrer 180-W-XPS™-GLL wieder im Ev. Krankenhaus Witten hospitalisiert. Das mittlere Alter dieser Patienten betrug 74,3 Jahre. Mittels einer logistischen Regressionsanalyse wurde für das Alter ein *p*-Wert von 0,0157 errechnet. Dies impliziert, dass ein Alter oberhalb des mittleren Alters einen statistischen, signifikanten Einfluss auf das Vorhandensein von Komplikationen hat.

### ASA-Score

Die von der American Society of Anesthesiologists (ASA) vorgeschlagene Klassifikation teilt die Patienten vor der Narkose anhand von systemischen Erkrankungen verschiedenen Risikogruppen bezüglich ihres körperlichen Zustands zu und geht aus Tab. 1 hervor [9].

**Table 1:** 

ASA-Klassifizierung	ASA-Score 1	ASA-Score 2	ASA-Score 3	ASA-Score 4
Patienten (%)	4,6	39,6	54,8	1

Aus der Regressionsanalyse ergibt sich eine 5,17fach erhöhte Wahrscheinlichkeit, von einer oder mehreren Komplikationen betroffen zu sein, für einen Patienten mit einem ASA-Score von 2 Punkten gegenüber einem Patienten mit einer ASA-1-Klassifikation und analog eine 24fach erhöhte Wahrscheinlichkeit bei einem ASA-Score von 3 vs. 1.

### Umfang der 180-W-XPS™-GLL

Der Mittelwert der Laser-Energieabgabe als Surrogatparameter für die Eingriffsdauer bzw. dem Umfang des Eingriffs lag bei 216.189 J. Der errechnete *p*-Wert für die Energieabgabe des Lasers in der Gegenüberstellung der Komplikationspatienten und Patienten ohne Komplikationen lag bei 0,475. Somit existiert kein signifikanter Unterschied in diesem Punkt.

### Komplikationsart

Wegen eines Harnverhalts wurden 85 Patienten mit Komplikationen oder 50,60 % aufgenommen, bei 83 Patienten oder 49,40 % handelte es sich um eine Hämaturie als Wiederaufnahmegrund.

Bei den 48 (28,24 %) mit einem Harnwegsinfekt wiederaufgenommenen Patienten wurde im Rahmen des Aufnahmeprozederes eine Urinkultur angelegt. Mit dieser gelang bei 29 (17,06 %) Patienten ein Erregernachweis (s. Tab. 2).

**Table 2:** 

Gram-negative Bakterien	*n*	%	Gram-positive Bakterien	*n*	%
Escherichia coli	10	25,8	Enterococcus faecalis	6	3,35
Pseudomonas aeruginosa	5	2,94	Aerococcus urinae	2	1,18
Proteus mirabilis	4	2,35	Staphylococcus aureus	1	0,59
Klebsiella pneumoniae	1	0,59			

### Antibiotikaregime

Die Betrachtung der Komplikationsrate bezüglich der Infektionen in den beschriebenen Zeiträumen 2012–2018 und ab 2019 mit dem Fisher-Test zeigten einen *p*-Wert > 5 %, sodass die Odds Ratio nicht signifikant von 1 verschieden ist. Damit hat die Art der Antibiotikatherapie keinen Einfluss auf das Vorhandensein von Komplikationen.

### Blutverdünnende Medikation

**Figure 2:**
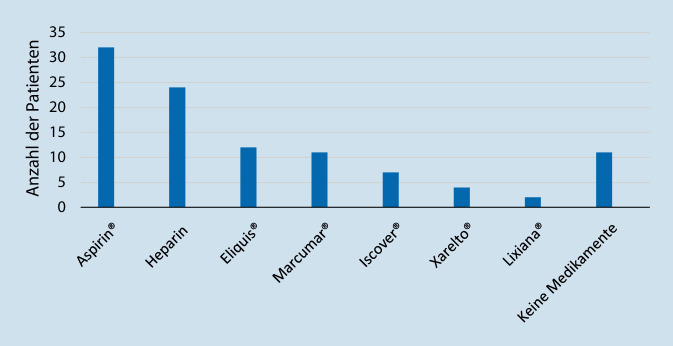

In der Abb. 2 ist die Häufigkeit der eingenommenen blutverdünnenden Medikamente bei Patienten mit Hämaturie dargestellt. Die gerinnungsaktiven Medikamente lassen sich in Thrombozytenaggregationshemmern (TAH/Plättchenhemmern) mit Acetylsalicylsäure (ASS® oder Aspirin®) und Clopidogrel (Iscover®), in Vitamin-K-Antagonisten (Phenprocoumon [Marcumar®]), sowie in die neuen oralen Antikoagulanzien (NOAK) einteilen. Zu diesen NOAK gehören Xarelto®, Eliquis® und Lixiana®. Von den 83 Patienten, die wegen einer Hämaturie wiederaufgenommen werden mussten, nahmen alle bis auf 11 (13,25 %) eines der betrachteten Medikamente.

## Diskussion

Erstmals überhaupt wurde in der vorliegenden Arbeit eine neue Systematik zur Erfassung von Komplikationen der 180-W-XPS™-GLL gewählt. Es wurden nicht die Frühkomplikationen der GLL, die im initialen Krankenhausaufenthalt oder in Studienzentren unter ambulanten Bedingungen bei systematischen Nachuntersuchungen auftraten, erfasst, sondern es standen die zu einer Wiederaufnahme führenden Komplikationen in einem 3‑Monats-Intervall im Fokus des Interesses.

Neben der Real-world-Betrachtung bietet diese Herangehensweise den Vorteil, dass nur schwerwiegendere Komplikationen herausgefiltert wurden, die nicht in einem ambulanten Setting behandelt werden konnten. Zu den Limitationen gehört, dass Wiederaufnahmen in Krankenhäuser außerhalb der operierenden Klinik nicht erfasst wurden. Die genannte Systematik trägt einer Besonderheit des häufig mit dem minimal-invasiven, besonders für multimorbide Patienten geeigneten Laserverfahrens Rechnung: Da es sich häufig um immobile, hochbetagte und in institutionalisierter Pflege lebende Patienten handelt, gestaltet sich gerade außerhalb von Praxisöffnungszeiten eine ambulante Vorstellung bei neu aufgetretenen Beschwerden schwierig. Werden diese Patienten in der operierenden Klinik vorgestellt, stellt sich (etwa nach möglicher ambulanter Behandlung) eine erneute Entlassung in ambulante Versorgung durch den behandelnden Krankenhausarzt in Anbetracht der besonderen Gebrechlichkeit und Vulnerabilität des geriatrischen Patienten als schwierig dar, sodass diese Patienten dann auch hospitalisiert werden. So konnte die Arbeitsgruppe Uro-Geriatrie der Universität Witten/Herdecke nachweisen, dass mit dem ISAR(„identification of seniors at risk“)-Screening als „geriatrisch“ markierte Patienten in der stationären Urologie signifikant häufiger als Notaufnahme in das Krankenhaus aufgenommen werden, als gleichaltrige Patienten ohne geriatrischen Handlungsbedarf (36 vs. 20,3 %, *p* = 0,001, *n* = 332; [8]).

Damit stellt nicht nur die Behandlungspflichtigkeit der Komplikationen insgesamt, sondern die Wiederaufnahme eine volkswirtschaftlich potenziell bedeutsame Größe dar. In Anbetracht der Eigenschaften der mit der 180-W-XPS™-GLL behandelten gebrechlichen, multimorbiden und multimedizierten Patienten ist der prozentuale Anteil, der innerhalb von 3 Monaten nach der Behandlung wiederaufgenommenen Patienten mit 13,25 % als vertretbar einzuschätzen.

Hier ist zu berücksichtigen, dass mehr als die Hälfte der betrachteten Patienten mit 54,8 % in die Kategorie 3 der von der ASA inaugurierten Einteilung fielen und somit als „Patienten mit schwerer Allgemeinerkrankung“ galten. Dies erklärt bei einem hohen Anteil multimorbider Patienten im Studienkollektiv die Wiederaufnahmerate von 13,25 %. Der Vergleich dieser Wiederaufnahmerate mit der multizentrischen „GOLIATH-Studie“, welche als Zulassungsstudie als Goldstandard zur Beschreibung der Verfahrenseffektivität und -sicherheit gesehen wird, zeigt bei einer Gesamtkomplikationsrate Patienten ab Clavien-Dindo Grad II (pharmakologische Therapie, Transfusion, parentale Ernährung) mit 14,07 % [3] vergleichbare Ergebnisse [10]. In der GOLIATH-Studie wurden alle Patienten mit einer Clavien-Dindo-Grad-I-Komplikation von der Analyse ausgeschlossen. Komplikationen dieser Art und ihre erforderliche konservative Therapie könnten jedoch bei multimorbiden, gebrechlichen Patienten durchaus zu Krankenhauseinweisungen etwa zum Ausgleich einer Elektrolytstörung oder bei Fieber führen, sodass die wahre Komplikationsrate hier noch höher liegen dürfte. Außerdem handelte es sich bei der genannten Untersuchung um eine Zulassungsstudie mit ambulanten Vorstellungen in den Studienzentren nach 3 Wochen, 3 und 6 Monaten. Allein die hierfür erforderliche Mobilität und Compliance dürfte daher auch zu einem Ausschluss von geriatrischen, institutionalisierten Patienten und damit zu einem Bias geführt haben.

Die Gestaltung der perioperativen Antibiotikaprophylaxe war mehrfach Gegenstand der internen Diskussion und wurde auch im „Antibiotic Stewardship-Projekt“ thematisiert. Es erfolgte schließlich die Umstellung der tradierten antibiotischen Therapie von 2 × 250 mg Ciprofloxacin oral auf eine Cephalosporin-gestützte Single-shot-Prophylaxe. Der vorliegende Vergleich der beiden Therapiephasen im Fisher-Test zeigt keinen signifikanten Einfluss des perioperativen Antibiotikatherapieregime auf das Vorhandensein von Komplikationen bzw. auftretenden Infekten. Vor dem Hintergrund der prinzipiellen Vorteile einer Single-shot-Prophylaxe z. B. im Hinblick auf die Entwicklung von Resistenzen und das Nebenwirkungsprofil der Chinolone ist bei fehlenden Unterschieden in der Komplikationsrate damit retrospektiv die Entscheidung zum Wechsel des Antibiotikaregimes auch unter Betrachtung der Wirksamkeit als richtig zu erachten.

Auch der über die Gesamt-Energieabgabe bestimmte Umfang der 180-W-XPS™-GLL hatte in der statistischen Analyse ebenfalls keinen signifikanten Effekt auf die Komplikationsrate. Die Hypothese, dass eine höhere Energieabgabe und damit ein höherer Gewebeabtrag über die größere Wundfläche zu einer höheren Komplikationsrate führen könnte, muss damit anhand der vorliegenden Daten verworfen werden. Dieses Ergebnis unserer Arbeit steht im Gegensatz zu der im Begleittext in Abschn. 3.5.3 der S2e-Leitlinien zur Therapie des BPS geäußerten Feststellung, dass mit steigendem Prostatavolumen auch die Komplikations- und vor allem die Reinterventionsrate stiege [6] und muss daher anhand unserer Daten hinterfragt werden.

Insgesamt ist anhand der vorliegenden Daten von mehr als 1200 Prozeduren die 180-W-XPS™-GLL der Prostata als eine hoch effektive und komplikationsarme Behandlung des BPS mit besonderer Eignung für geriatrische Patienten unter Blutverdünnung zu sehen. Die Komplikationsrate erscheint im Hinblick auf die Multimorbidität der behandelten Patienten vertretbar, macht aber ein sorgfältiges Entlassmanagement mit der Organisation einer poststationären Versorgung und hier insbesondere mit einer Verzahnung des ambulanten mit dem stationären Sektor gerade für den Fall des Auftretens von Komplikationen notwendig. Dies gilt nach den vorliegenden Daten insbesondere dann, wenn es sich um besonders hochbetagte Patienten mit Blutverdünnung handelt.

## Fazit für die Praxis

- Eine bilaterale Kommunikation zwischen Urologen und Hausarzt über Art und Umfang der stattgehabten Operation und ihrer speziellen Risiken sollte eingeführt werden.
- Kontrollen des Urins auf Infektfreiheit postoperativ bzw. eine eventuelle Therapie einer detektierten Harnwegsinfektion auch bei Patienten in ambulanter oder stationärer Pflege sollte organisiert werden.
- Dauer einer NOAK(neue orale Antikoagulanzien)- oder Phenprocoumon-Pause sollte nach gemeinsamer Absprache erfolgen.
- „Notfallpläne“ für den Fall des Auftretens einer möglichen Komplikation müssten entwickelt werden.
- Die Information bzw. Schulung von Betreuungspersonen über Art und Umfang der durchgeführten Operation, der möglichen Risiken und Verhaltensmaßregeln ist sehr wichtig.
